# Auditory brainstem responses in the red-eared slider *Trachemys scripta elegans* (Testudoformes: Emydidae) reveal sexually dimorphic hearing sensitivity

**DOI:** 10.1007/s00359-019-01372-y

**Published:** 2019-10-25

**Authors:** Tongliang Wang, Handong Li, Jianguo Cui, Xiaofei Zhai, Haitao Shi, Jichao Wang

**Affiliations:** 1 Ministry of Education Key Laboratory for Ecology of Tropical Islands, College of Life Sciences, Hainan Normal University, Haikou, China; 2 Chengdu Institute of Biology, Chinese Academy of Sciences, Chengdu, China

**Keywords:** Auditory brainstem response, Sexual dimorphism, Threshold, *Trachemys scripta elegans*, Tympanic membrane

## Abstract

Hearing sensitivity is of general interest from the perspective of understanding the functionality and evolution of vertebrate auditory systems. Sexual dimorphism of auditory systems has been reported in several species of vertebrates, but little is known about this phenomenon in turtles. Some morphological characteristics, such as middle ear and tympanic membrane that influence the hearing sensitivity of animals can result in hearing sexual dimorphism. To examine whether sexual dimorphism in hearing sensitivity occurs in turtles and to compare hearing characteristics with respect to the shape of the tympanic membrane, we measured the hearing sensitivity and tympanum diameter in both sexes of *Trachemys scripta elegans*. The results showed that, with the exception of 0.9 kHz, auditory brainstem response thresholds were significantly lower in females than in males for frequencies in the 0.2–1.1 kHz range, indicating that the hearing of females shows greater sensitivity. No significant differences were detected in the tympanum diameter of both sexes. These results showed that sexually dimorphic hearing sensitivity has evolved in turtles; however, this difference does not appear to be related to differences in the size of the tympanic membrane. The possible origin and function of the sexual differences in auditory characteristic are discussed.

## Introduction

Sexual dimorphism refers to differences in body size, shape, color, or other morphological features of the female and male individuals of the same species (Hedrick and Temeles 1989; Andersson 1994; Katsikaros and Shine 2010), which reflect differences in the adaptations of males and females. Sexual dimorphism can arise from the interaction between natural and sexual selection, which can act independently or interact synergistically, depending on various factors (Shine 1989; Arnold 1994; Djurakíc et al. 2011). At present, sexual selection (Lovich and Gibbons 1992; Willemsen and Hailey 2003), fecundity effort (Kupfer 2009; Olsson et al. 2010), and niche divergence (Thom et al. 2010) are the three major hypotheses that have been proposed to explain the evolution and maintenance of sexual size dimorphism (SSD). Although most studies on sexual dimorphism have concentrated on body size or shape, sexually dimorphic hearing sensitivity has also been reported in some species of amphibians (Narins and Capranica 1976; Wang et al. 2016, 2019; Yang et al. 2018). Remarkably, such sexually dimorphic hearing sensitivity may primarily result from sexual dimorphism of the auditory organs (particularly tympanic membranes) and their physical properties (Dijk et al. 2002; Feng et al. 2006; Shen et al. 2011; Liu et al. 2014; Wang et al. 2016).

Chelonian turtles are a monophyletic group of reptiles that occupy a wide range of ecological niches from the desert to the ocean, among which sexual dimorphism is a common feature, with the females of most species being larger than the males (Willemsen and Hailey 2003; Lefebvre et al. 2011). Most studies on sexual dimorphism in chelonians that have either examined multiple species (Willemsen and Hailey 2003; Kaddour et al. 2008) or different geographical populations of a single species which focused on body size (Gibbons and Lovich 1990; Yasukawa et al. 1996; Djordjević et al. 2013). Although it has been determined that hearing plays an important role in the survival, reproductive success, and numerous social behaviors of chelonians that use acoustic communication (Ferrara et al. 2013, 2014; Köppl et al. 2014), the sexual differences in hearing, which may well be a common form of sexual dimorphism, are currently not well understood (Ferrara et al. 2014; Willis 2016). Hearing sensitivity is of general interest with respect to gaining an understanding of the functionality of auditory systems (Christensen-Dalsgaard et al. 2012), and the study of sexual dimorphism in hearing sensitivity has become increasingly important as it has provided insights on the functional differences in the auditory systems of male and female turtles.

The red-eared slider (*Trachemys scripta elegans*) is a semi-aquatic freshwater turtle that is native to the eastern United States and northeastern Mexico. Owing to its high ecological tolerance and behavioral plasticity, this species of slider has been listed as one of the 100 most successful invasive species in the world (Lowe et al. 2000; Kraus 2009; Ma and Shi 2017). *T. scripta elegans* has also been observed to exhibit SSD, with females having larger body sizes than males (Gibbons and Lovich 1990). Furthermore, based on spectral analysis and visual modeling, Wang et al. (2013) observed significant differences in hues of the ultraviolet (UV) components of body colors in male and female *T. scripta elegans*.

Studies investigating the hearing of *T. scripta elegans* have revealed that this species is more sensitive to sound underwater than in the air, and that this sensitivity depends on their large middle ear, which is characterized by a compliant tympanic disc that is attached to the columella (Christensen-Dalsgaard et al. 2012). The tympanic membrane is the primary sound-receiving structure of the turtle ear (Wever 1978; Christensen-Dalsgaard et al. 2012). The origin and morphology of the ear, as well as the mechanism underlying the transduction of sound into neural signals via hair cells, have previously been described in detail (Hackney et al. 1993; Clack 1997). To the best of our knowledge, there have to date been no comparative studies on hearing sensitivity in male and female turtles with tympanic membranes. In this study, we measured the hearing sensitivity and auditory functionality of both sexes of *T. scripta elegans* using auditory brainstem responses (ABRs), the measurement of which has been verified as a non-invasive and rapid method for determining hearing sensitivity and auditory system functionality (Walsh et al. 1992; Brittan-Powell et al. 2010). We also sought to determine relationships between hearing characteristics and the morphological characteristics of the tympanic membrane. Given that the body size of females is larger than that of males, we predicted that the diameter of the tympanic membrane in females would be larger than that of males, and that hearing would thus be more sensitive in females.

## Materials and methods

### Animals

As experimental animals in this study, we used 5-year-old male (*n* = 10) and female (*n* = 10) *T. scripta elegans*. The morphological data of all individuals are shown in Table 1. All animals were purchased from farms in Hainan Province, and were maintained in standard aquaria at 28–32 **°**C prior to the experiments. Prior to electrode placement, each turtle was deeply anesthetized using a solution of 0.5% pelltobarbitalum natricum (Xiya Reagents, Shandong, China) dissolved in 0.9% sodium chloride. The anesthetic was administered via hind limb intramuscular injection at an initial dose of 0.003 mL g^−1^. Additional doses (each at 20% of the initial dose) were administered in cases when the subject was not deeply anesthetized. Electrophysiological experiments commenced when the subject showed no reflexive response to stimulation of the hind leg muscles and eyes with a pair of forceps. The animals remained relatively motionless for up to 150 min. Having completed data collection, the turtle was placed in a bucket without water and allowed to recover from sedation. Once the animal was fully revived and moving, it was returned to a culture pond in our laboratory. The animal treatment procedures were approved by the Animal Research Ethics Committee of Hainan Provincial Education Centre for Ecology and Environment, Hainan Normal University (HNECEE-2018-001), and were carried out in strict compliance with the institutional guidelines.

**Table 1:** Comparison of differences in the morphological traits of *Trachemys scripta elegans* between the sexes

Parameters	Sex		Statistical summary	
	Female (*n* = 10)	Male (*n* = 10)	*F*	*p*
Body mass (g)	1681.60 ± 195.31	1069.7 ± 151.06	61.41	< 0.001
Carapace length (mm)	223.56 ± 9.36	197.01 ± 11.17	33.20	< 0.001
Tympanum diameter (mm)	10.63 ± 1.41	9.64 ± 0.90	3.49	0.08

The variance is presented in terms of the mean ± standard deviation

### Auditory brainstem response (ABR) measurements

All recordings took place in a sound-proof booth lined with echo-attenuating acoustic foam. ABRs were recorded for approximately 100 min, during which time the turtles remained anesthetized. The turtles were positioned so that the speaker (frequency response range 55 Hz–20 kHz, JBL GT7-6, Harman International Industries, Inc., Stamford, CT, USA) was 5 cm from the tip of the turtle’s jaw. Standard platinum alloy, subdermal needle electrodes (27 ga, 13 mm in length, Rochester Electro-Medical, Inc., Lutz, FL, USA) were inserted subdermally above the tympanum (recording electrode), on the top of the head under the frontal scale (reference electrode), and in the ipsilateral front leg (ground electrode). Electrode impedance was less than 3 kΩ.

Stimulus presentation, ABR acquisition, equipment control, and data management were coordinated using a TDT RZ6 Multi-I/O Processor, linked via fiber optic cables to a TDT RA4LI low-impedance digital headstage and RA4PA Medusa preamp and linked to a laptop computer running BioSig software (Tucker-Davis Technologies, Alachua, FL, USA). Sound stimuli were generated using a TDT RZ6 Multi-I/O Processor, which directly drove the speaker while running TDT SigGen software. As shown in multiple previous studies, two types of signals as acoustic stimuli (tone bursts and broadband clicks) were used in this study (Brittan-Powell et al. 2002, 2010). Broadband clicks generally result in well-formed responses and can be obtained in a relatively short time interval (Gorga et al. 2006). Tone burst (9 ms duration, 2 ms rise/fall time, with a sample rate of 24,414 Hz and an alternating polarity) was synthesized digitally from 0.2 to 1.5 kHz in 100 Hz increments, attenuated in 5 dB steps from 90 to 35 dB sound pressure level (SPL), and presented at a rate of 4/s. Clicks were 0.1 ms in duration with a 249 ms interstimulus interval, attenuated in 5 dB steps from 90 dB to 35 dB SPL, and presented at a rate of 4/s. The clicks exhibited a nearly flat power spectrum of approximately 53 dB between 1 and 2000 Hz. Each ABR wave represented the average response to 200 stimulus presentations. Signals from the electrodes were amplified (20×), filtered (high pass 30 Hz; low pass 3 kHz; notch filtered 50 Hz). Sound stimulus was played a single time from low to high frequency.

ABR stimulus levels were calibrated in the free field using a sensor signal conditioner (model 480C02, PCB Piezotronics, Inc., Depew, NY, USA) with a 1/4″ microphone (model 426B03 PCB Piezotronics, Inc., Depew, NY, USA) approximately positioned at the tip of the jaw of the turtle, but with the turtle absent. The distance between the speaker and the tip of the turtle jaw was fixed at 5 cm and calibrated using a TDT RZ6 Multi-I/O Processor and BioSigRP (Tucker-Davis Technologies, Inc., Florida, USA), which repeatedly played the signal at the same rate used while recording ABRs and simultaneously recorded the microphone signal at a sampling rate of 24,414 Hz.

The ABR thresholds and latencies were determined using visual inspection similar to that described by Brittan-Powell et al. (2002). Threshold measurements were defined as the lowest stimulus level for which no repeatable responses could be recognized (Zhang et al. 2012; Bierman et al. 2014; Scott et al. 2017). To reduce inter-rater variability, all turtle ABR thresholds were determined by the same experienced person. We assumed that the 80 dB level was higher than the ABR thresholds of all turtles included in this study for the stimuli used.

### Measurement of morphological data

**Fig. 1:**
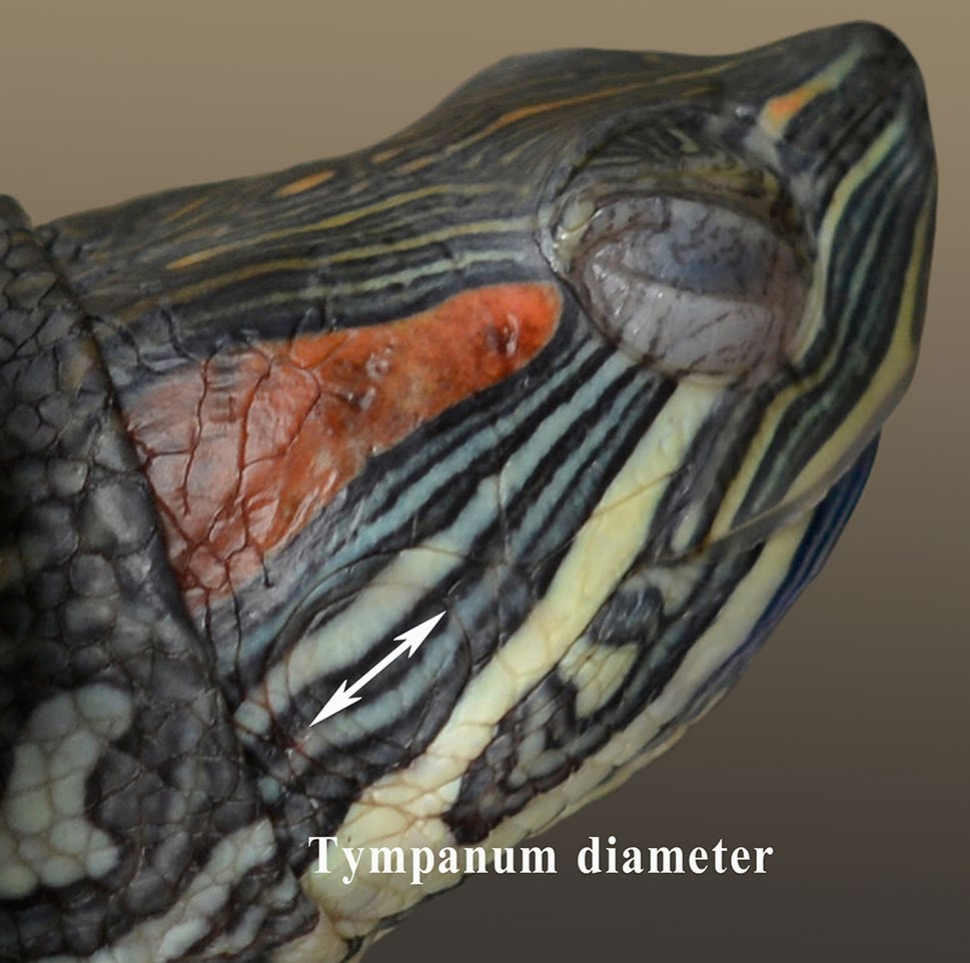
Measurements of the diameter of the tympanum from a male turtle. Inset: schematic diagram showing how the tympanum diameter was measured

Prior to recording ABRs, the body mass of all turtle specimens was recorded using an electronic balance [SI-234, Denver Instrument (Beijing) Co., Ltd., Beijing, China], whereas the tympanum diameter (Fig. 1) and carapace length were measured using a Mitutoyo digital caliper (500-196-30, Mitutoyo Corp., Kawasaki-shi, Kanagawa Prefecture, Japan).

### Analysis and statistics

The ABR thresholds and latencies obtained from female and male *T. scripta elegans* in response to tone and click stimuli were sorted and analyzed using SPSS 22.0 software (IBM Corp., Chicago, IL, USA). Prior to statistical analysis, all data were examined for assumptions of normality and homogeneity of variance, using the Shapiro–Wilk and Levene tests, respectively. Data on ABR thresholds, ABR latency, and ABR amplitude of both sexes were analyzed using a repeated-measures factorial ANOVA and one-way ANCOVA. The body mass, carapace length, and tympanum diameter of both sexes were analyzed using one-way ANCOVA. Results are expressed as the mean ± SD, and a *p* value < 0.05 was considered to indicate significant difference.

## Results

### Auditory brainstem response (ABR) wave morphology

**Fig. 2:**
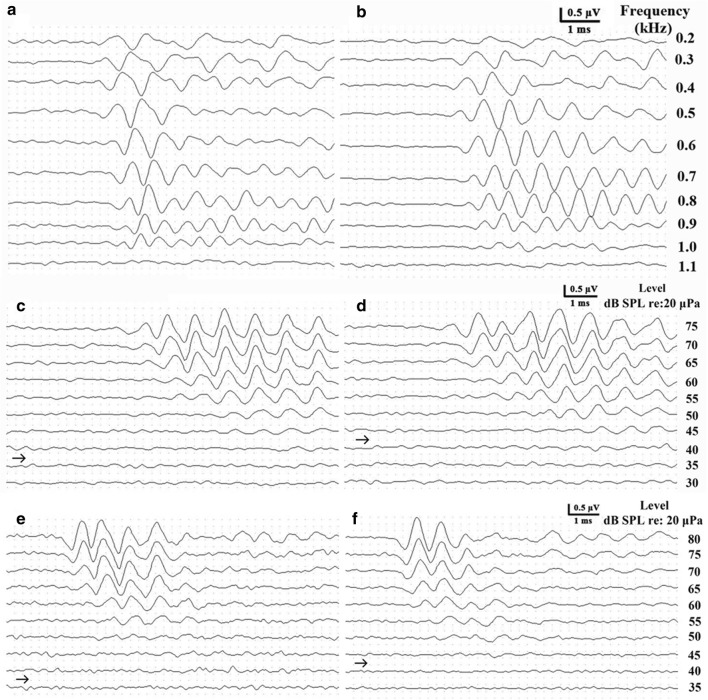
Auditory brainstem responses (ABRs) elicited in response to frequency-specific tone bursts at a 70-dB sound pressure level, showing valley–peak waveforms from a female (**a**) and a male (**b**) *Trachemys scripta elegans*. ABRs as a function of stimulus intensity evoked by a tone burst of 0.6 kHz from a female (**c**), a male (**d**) and by a click stimulus from a female (**e**) and a male (**f**) *T. scripta elegans*. ABRs as a function of intensity evoked in *T. scripta elegans*. The right-pointing arrowheads indicate the visually detected thresholds for each stimulus frequency

In both females and males, the ABR to tone burst (Fig. 2a–d) and click (Fig. 2e, f) stimuli were characterized by valley–peak waveforms; however, in both females and males, the waveforms were not obvious at or above 1.1 kHz (Fig. 2a, b). The dominant valleys and peaks were clearly visible in all waveforms (Fig. 2c–f).

### Auditory brainstem response (ABR) thresholds

**Fig. 3:**
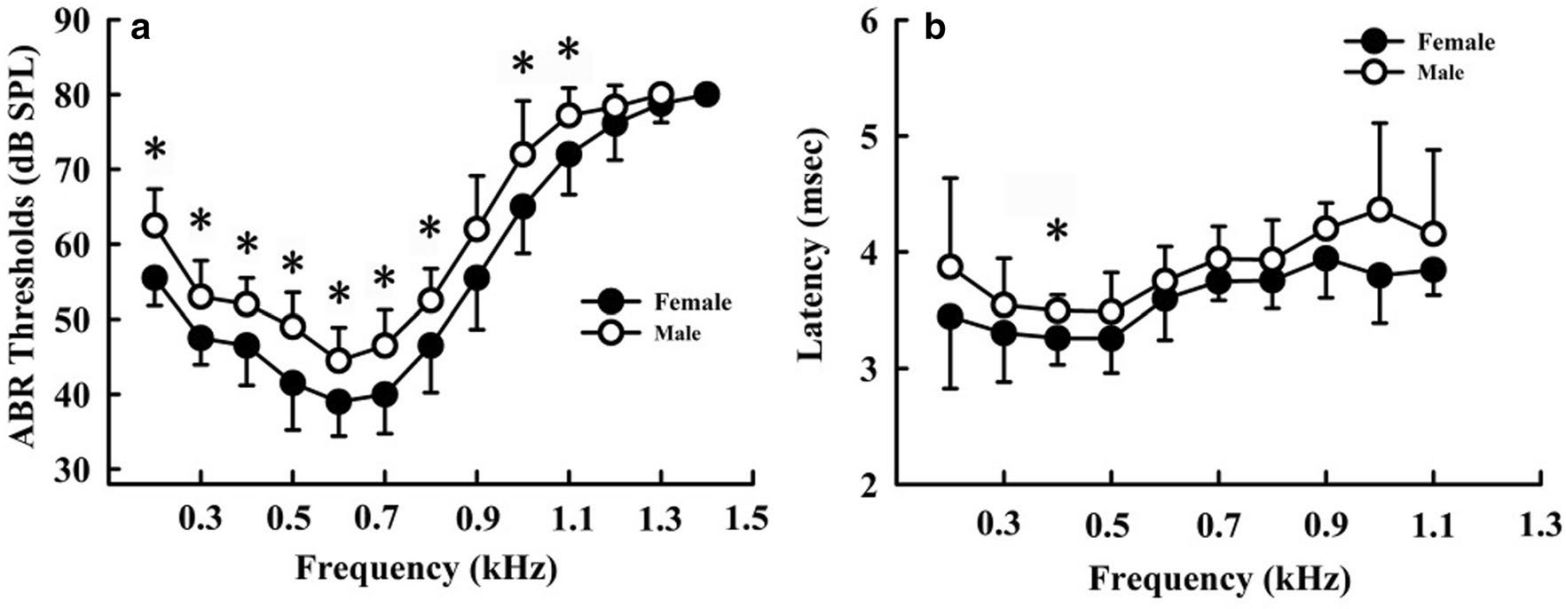
Auditory brainstem response (ABR) thresholds (**a**) and latency (**b**) in female and male *Trachemys scripta elegans*. The plotted points represent the thresholds or latency for tone bursts (mean ± standard deviation). *p < 0.05

We measured the ABR thresholds of individual turtles at all predetermined stimulus frequencies. Thereafter, the ABR thresholds of each stimulus frequency were compared between females and males. We observed clear threshold differences between females and males. Figure 2c, d shows a typical ABR response level series measured in one female and one male, for which thresholds of 40 dB SPL and 45 dB SPL were obtained at 0.6 kHz, respectively. There were significant differences in ABR thresholds between frequencies (*F* = 131.23, *df* = 8, *p* < 0.001) and sex (*F* = 11.16, *df* = 1, *p* = 0.004), whereas we detected no significant interactive effect between sex and frequency (*F* = 0.24, *df* = 8, *p* = 0.98) (Fig. 3a). As shown in Fig. 3a, with the exception of thresholds at 0.9 kHz (*p* = 0.053), the ABR thresholds of females were significantly lower than those of males from 0.2 to 1.1 kHz (*p* < 0.05). Although differences in the thresholds of females and males were not statistically significant between at 0.9 kHz, they were lower for females compared to males. Across the 0.2–1.1 kHz frequency range, tone burst thresholds were generally approximately 3 dB lower in females than in males.

### Auditory brainstem response (ABR) latency

ABR latencies were measured between stimulus onset and the waveform valley. We selected 75 dB for all stimulus frequencies to determine ABR latency, and then the ABR latency of each stimulus frequency was compared between females and males. We detected no significant interactive effect between sex and latency (*F* = 3.91; *df* = 1, *p* = 0.06), although there were differences between females and males at 0.4 kHz (*p* = 0.012) (Fig. 3b).

### Auditory brainstem response (ABR) amplitude

**Fig. 4:**
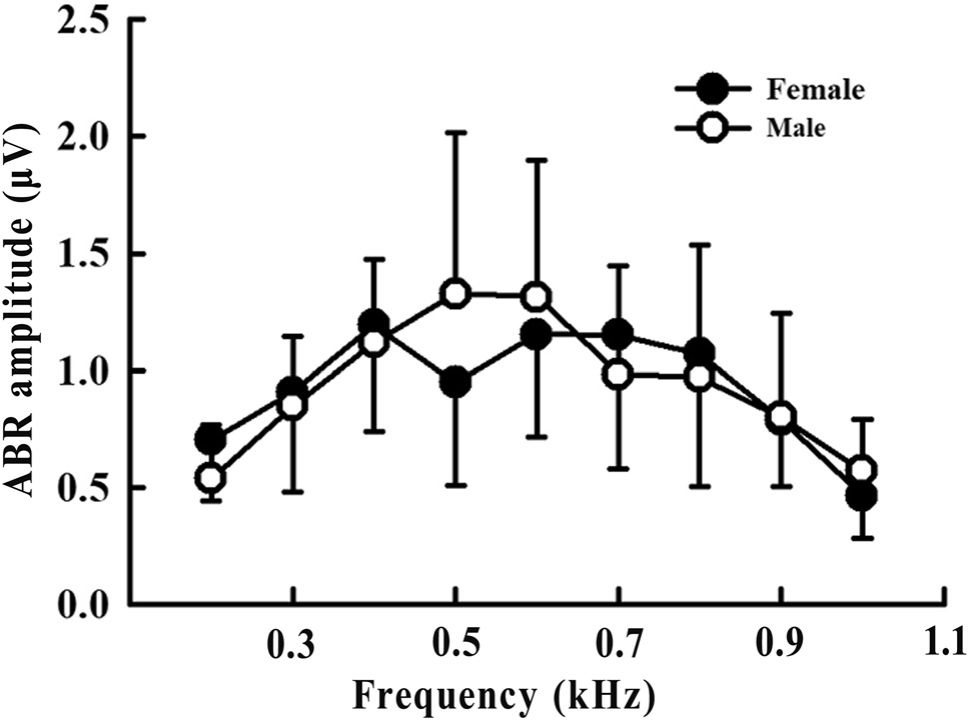
Auditory brainstem response (ABR) amplitude in female and male *Trachemys scripta elegans*. The plotted points represent the ABR amplitude for tone bursts (mean ± standard deviation)

Auditory brainstem response (ABR) amplitude (absolute voltage difference between P1 and N1) was obtained from 75 dB at all stimulus frequencies, and the ABR amplitude of each stimulus frequency was compared between females and males. As shown in Fig. 4, there was no significant difference in the ABR amplitudes of females and males (*p* > 0.05).

### Morphological data

Data relating to body mass, tympanum diameter, and carapace length are presented in Table 1. Both body size and carapace length were significantly larger in females than in males (*p* < 0.001), whereas there was no significant difference between the sexes with regard to tympanum diameter (*p* = 0.08).

## Discussion

Although calls have been verified to be an important communication method for chelonians (Galeotti et al. 2005; Ferrara et al. 2013, 2014), and play an indispensable role in survival, reproduction, and other social behaviors (Galeotti et al. 2004; Giles et al. 2009), there have been comparatively few studies on the hearing sensitivity of chelonians (Martin et al. 2012; Köppl et al. 2014). To the best of our knowledge, this is the first study in which the hearing sensitivity of both sexes of a single turtle species has been compared. We found that female and male *T. scripta elegans* have a similar range of sensitivities (0.2–0.9 kHz), whereas the ABR threshold of females was significantly lower than that in males. These results provide convincing evidence that sexually dimorphic hearing sensitivity has emerged in turtles, and that the hearing of females is more sensitive than that of males, which is consistent with previous findings for frogs and toads (Wang et al. 2016, 2019; Yang et al. 2018).

Sexual dimorphism could arise from ecological differences between females and males, from natural selection for fecundity or parental care, or from sexual selection for courtship success (Shine 1989; Andersson 1994; Willemsen and Hailey 2003). Sexual selection is believed to be one of the main factors that explains the evolution of sexually dimorphism in some species of reptiles (Kratochvíl and Frynta 2010). The size when each sex attains maturity is the underlying factor promoting SSD in *T. scripta elegans*, and it is the critical life history trait upon which natural and sexual selection have operated to determine the ultimate SSD observed (Gibbons and Lovich 1990). The more sensitive hearing of females may contribute to an enhancement of predation efficiency, thereby enabling females to reach maturity with larger body mass, to reduce the risk of predation, and to enable females to meet the expensive energy demand associated with reproduction. More importantly, given that female turtles spend more time on land than males during the reproductive period, when incubating and laying eggs, sensitive hearing may have enabled them to adapt more effectively to the complex terrestrial environment. Although the behavioral significance of sexually dimorphic hearing sensitivity in turtles remains to be determined, we suggest that both natural selection and sexual selection may have contributed to the evolution and maintenance of this dimorphism in turtles.

Previous studies have shown that the hearing sensitivity of *T. scripta elegans* is affected by the middle ear (Christensen-Dalsgaard et al. 2012). Moreover, several studies have suggested that a variety of morphological characteristics (including body size, middle ear structure, and tympanic membrane) influence the hearing sensitivity of fish and frogs (Christensen-Dalsgaard and Elepfandt 1995; Yan et al. 2000). In the present study, we detected no difference in the tympanum diameter of female and male *T. scripta elegans*, indicating that the size of the tympanic membrane may not be correlated with the size of the middle ear, and it is not directly related to the sexually dimorphic hearing sensitivity observed in turtles. However, research in frogs has indicated that differences in the hearing sensitivity may be linked to differences between the sexes in the size of the tympanic membrane, and the resulting differences in the size of the middle ear cavity (Feng et al. 2006). Accordingly, the hypothesis that sexually dimorphic hearing sensitivity in female and male *T. scripta elegans* results from differences in the size of the middle ear needs to be further examined in the future.

Most anurans possess a tympanic middle ear that is sensitive to airborne sound, which is processed by the amphibian and basilar papillae (Manley et al. 2004; Christensen-Dalsgaard 2009). In turtles, the auditory papilla is small and, similar to all amniote papillae, organized tonotopically, such that higher frequency sounds excite the hair cells at the base and lower frequencies those at the apex (Crawford and Fettiplace 1980). The hearing range of the female and male *T. scripta elegans* has been found to be confined to low frequencies and does not differ significantly between the sexes, which may be because neither sex possesses a high-frequency region in their papillae (Manley 2010; Köppl et al. 2014).

Previous research has reported that juvenile green sea turtles (*Chelonia mydas*) detect acoustic stimuli in aerial stimuli between 0.05 and 0.8 kHz, with a maximum sensitivity of between 0.3 and 0.4 kHz in air (Piniak et al. 2016). In juvenile loggerhead sea turtles (*Caretta caretta*), this sensitivity is at least 0.25–0.75 kHz (Bartol et al. 1999). We found that the hearing range of *T. scripta elegans* lies between 0.2 and 0.9 kHz, with lowest thresholds of approximately 40 dB SPL of 0.6 kHz in females and 45 dB SPL of 0.6 kHz in males. These results further confirm that the hearing range of Chelonians is confined to low frequencies (mostly below 1 kHz). However, Christensen-Dalsgaard et al. (2012) have reported that the ABR audiogram of *T. scripta elegans* in air indicates the highest sensitivity to sound at 0.3–0.5 kHz and lowest thresholds at approximately 60 dB SPL. Compared with the findings of the present study, there are obvious differences in both the hearing range and thresholds reported by Christensen-Dalsgaard et al. (2012). In our analysis, we found that the body mass of the turtles (female: 1681.60 ± 195.31 g, male: 1069.7 ± 151.06 g) was smaller than that of the specimens (150–500 g) examined by Christensen-Dalsgaard et al. (2012), who averaged the results from both sexes and therefore did not account for the influence of sexually dimorphic hearing sensitivity. Although no statistically significant differences have been detected in the scaling of the volume of the bony middle ear cavity with head size among most chelonian families when categorized by phylogeny and ecology (Willis et al. 2013), we still speculate that changes in body mass from the juvenile stage to adulthood may be an important life history trait that influences the hearing sensitivity of turtles. Consequently, the phenomenon and mechanisms of the age-dependent plasticity of sexually dimorphic hearing sensitivity require additional research in the future.

## Abbreviations

ABR: Auditory brainstem response
SSD: Sexual size dimorphism
SPL: Sound pressure level
